# The Proof‐of‐Concept of Anode‐Free Rechargeable Mg Batteries

**DOI:** 10.1002/advs.202207563

**Published:** 2023-03-20

**Authors:** Minglei Mao, Xueru Fan, Wei Xie, Haoxiang Wang, Liumin Suo, Chengliang Wang

**Affiliations:** ^1^ School of Integrated Circuits, School of Optical and Electronic Information, Wuhan National Laboratory for Optoelectronics (WNLO) Huazhong University of Science and Technology Wuhan 430074 P. R. China; ^2^ Beijing Advanced Innovation Center for Materials Genome Engineering, Institute of Physics Chinese Academy of Sciences/Beijing National Laboratory for Condensed Matter Physics Beijing 100190 P. R. China; ^3^ Wenzhou Advanced Manufacturing Institute Huazhong University of Science and Technology 325035 Wenzhou P. R. China

**Keywords:** adsorption, anode‐free, electrocatalysis, rechargeable Mg batteries, volumetric energy density

## Abstract

The desperate pursuit of high gravimetric specific energy leads to the ignorance of volumetric energy density that is one of the basic requirements for batteries. Due to the high volumetric capacity, less‐prone formation of dendrite, and low reduction potential of Mg metal, rechargeable Mg batteries are considered with innately high volumetric energy density. Nevertheless, the substantial elevation in energy density is compromised by extremely excessive Mg metal anode. Herein, the proof‐of‐concept of anode‐free Mg_2_Mo_6_S_8_‐MgS/Cu batteries is proposed, in which MgS as the premagnesiation additive constantly decomposes to replenish Mg loss by electrolyte corrosion over cycling, while both Mg_2_Mo_6_S_8_ and MgS acts as the active material to reversibly provide high capacities. Besides, Mg_2_Mo_6_S_8_ shows superior catalytic activity on the decomposition of MgS and provides the strong affinity to polysulfides to restrain their dissolution. Consequently, the anode‐free Mg_2_Mo_6_S_8_‐MgS/Cu batteries deliver a high reversible capacity of 190 mAh g^−1^ with the capacity retention of 92% after 100 cycles, corresponding to the highly competitive energy density of 420 Wh L^−1^. The proposed anode‐free Mg battery here spotlights the great promise of Mg batteries in achieving high volumetric energy densities, which will significantly expedite the advances of Mg batteries in practice.

## Graphical abstract

Rechargeable Mg batteries are expected to achieve high volumetric energy densities only if the excess Mg is limited. Herein, the proof‐of‐concept of anode‐free Mg batteries is first proposed using Mg_2_Mo_6_S_8_‐MgS composite cathode. The anode‐free Mg_2_Mo_6_S_8_‐MgS/Cu batteries deliver a capacity of 190 mAh g^−1^ with capacity retention of 92% after 100 cycles, corresponding to the energy density of 420 Wh L^−1^.

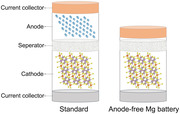

## 1 Introduction

Volumetric energy densities of rechargeable batteries, as one of the basic requirements for energy storage systems, are more or at least equally important than the gravimetric specific energy in some commercial applications, such as portable devices and electric vehicles where space is a major concern.^[^ ^1^ ^]^ Although ignored for a long period of time, volumetric energy densities have recently attracted the increasing attentions.^[^ ^2^ ^]^ Due to the high capacities and low potentials, metal anodes especially Li metal anode (LMA) are proposed to replace the intercalation anodes which are reaching the ceiling of capacities. Theoretically, replacing the graphite electrode with lithium metal will result in a ~50% elevation in volumetric energy density at the cell level.^[^ ^3^ ^]^ However, the implementation of lithium metal batteries (LMBs) is afflicted by low coulombic efficiency (CE) of Li deposition/stripping and safety hazards derived from Li dendrites.^[^ ^4^ ^]^ In addition, mossy Li generated during Li stripping/deposition process will cause high porosity of Li metal anode.^[^ ^5^ ^]^ 75% of the porosity of Li metal anode will completely compromise the elevation of volumetric energy densities at the cell levels.^[^ ^6^ ^]^ Therefore, a careful evaluation should be conducted to conclude the advantage of Li metal in enhancing volumetric energy densities of LMBs. Applying high pressure is proposed to control the porosity of Li metal,^[^ ^7^ ^]^ which however is not very pragmatic in practice. Instead, leveraging multivalent metal anodes with higher volumetric capacities, such as Mg, Ca, Zn, and Al,^[^ ^8^ ^]^ is considered as a practical alternative to circumvent the issues of Li metal.

Among the potential multivalent‐metal anodes, Mg metal stands out featuring high volumetric capacities (3833 mAh cm^−3^ vs. 2046 mAh cm^−3^ for Li), less propensity to form dendrites with high stripping/plating CE, as well as abundant reserve and low potential (−2.37 V vs. standard hydrogen electrode (SHE)).^[^ ^9^ ^]^ Paired with oxide or sulfide cathodes, rechargeable Mg batteries (RMBs) are expected to achieve high volumetric energy densities.^[^ ^10^ ^]^ Nevertheless, the promising energy density can be achieved only if the excess Mg is limited especially in zero‐excess configuration where volume is minimized and thus energy density maximized. Unfortunately, RMBs reported in the literature often use extremely excessive Mg metal containing over ten times the amount than that is actually cycled.^[^ ^11^ ^]^ The considerable excess could never be used in a practical cell and makes the interpretation of results more difficult as the cycling stability becomes artificially enhanced. Therefore, controlling Mg excess is imperative to develop practical and high‐energy‐density RMBs. However, limiting Mg excess is a big challenge, as Mg metal is constantly consumed by electrolyte corrosion during deposition/stripping process with CEs less than 100%.^[^ ^9^, ^12^ ^]^ Once Mg metal is drained, the capacity decay will be exacerbated. The deterioration of cycling stability will be accelerated in the zero‐excess or anode‐free configuration where cells are built with a bare current collect in the anode side and Mg is plated directly from the cathode in the first charge process.

**Figure 1:**
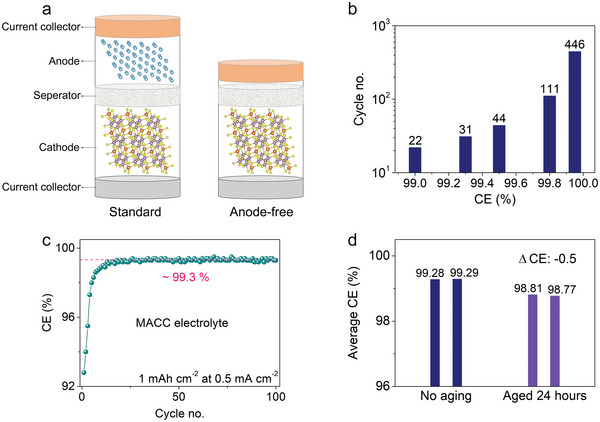
Effect of electrolyte on the cycle life of Mg batteries. a) Schematic illustrations of standard and anode‐free rechargeable Mg batteries. b) Relationship of the predicted cycle life of anode‐free Mg batteries with the Coulombic efficiency (CE) of Mg metal anode. Battery failure is defined when its capacity degrades to 80% of the original value. c) CE of Mg deposition/stripping process in Mg/Cu cells using MACC electrolyte for 1 mAh cm^−2^ at 0.5 mA cm^−2^. d) Effect of the 24 h interval resting on the average CE of Mg deposition/stripping process.

The predicted cycle life of anode‐free Mg batteries versus CE of Mg metal anode (MMA) is plotted (**Figure** **1**b). Putting aside cathode degradation, batteries are defined to fail when the capacity of MMA degrades to 80% of the original value. The cycle life is positively related with CE of MMA, in which Mg batteries only survive 22 cycles at a CE of 99%. Upon increasing the CE to 99.95%, the cycle life can be considerably extended to 446 cycles that can hardly yet meet the basic requirements of practical batteries for 500 cycles. Nonetheless in fact, it is quite challenging for Mg electrolyte to achieve a higher CE of Mg stripping/deposition than 99.9%.^[^ ^9^, ^13^ ^]^ As one of the typical electrolytes, Mg and Al chloride complex (MACC) electrolyte can provide a high CE of 99.3% over 100 cycles (Figure 1c) and long‐term dendrite‐free cycle of Mg metal (Figure S1–S3, Supporting Information), and is therefore adopted in this work. As the 99.3% CE of MACC electrolyte can only support 31 cycles (Figure 1b), to enable the practicable cycle life of anode‐free rechargeable Mg batteries (AFRMBs), the premagnesiation strategy is necessary to replenish fresh Mg over cycling.

Generally, there are two ways to compensate for the Mg loss: anode and cathode premagnesiation additives. Due to the low potentials versus Mg/Mg^2+^ at open circuit, the anode premagnesiation additives including Mg metal, though with high Mg‐compensation efficiency, are challenged by the high chemical reactivity and instability under ambient and battery fabrication conditions, as well as being not compatible with the regular industrial slurry‐based electrode fabrication using *N*‐methyl‐pyrrolidone as the solvent.^[^ ^14^ ^]^ Worse still, the reduction of electrolyte at the surface of anode premagnesiation additives and the growth of solid electrolyte interphase (SEI) will cause galvanic corrosion of Mg during calendar ageing even in the discharged state.^[^ ^15^ ^]^ To evaluate effects of calendar aging in MACC electrolyte, Mg/Cu cells are aged for 24 h at open circuit after the plating step (Figure 1d and Figure S4, Supporting Information). The average CE decreases by ≈0.5% after 24 h of calendar ageing. The difference between the CE with and without calendar ageing quantifies the extra loss of capacity. The unneglectable capacity loss in a short calendar ageing time of 24 h undermines the application of anode premagnesiation additives in our anode‐free RMBs.

**Figure 2:**
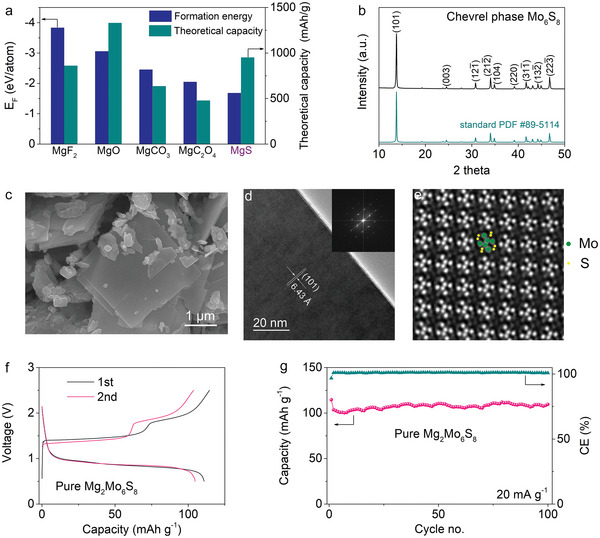
Screening cathode premagnesiation additives and performance of Mg_2_Mo_6_S_8_ cathode. a) Comparison in the formation energy and theoretical capacity of cathode premagnesiation additives. b) X‐ray diffraction (XRD) patterns, c) scanning electron microscopy (SEM), and d) high‐resolution transmission electron microscopy (HRTEM) of Chevrel phase Mo_6_S_8_. Inset of (d) is the corresponding fast Fourier transform (FFT). e) STEM‐LAADF image of the single crystal of Mo_6_S_8_ along the [211] zone axis. The bright and gray spots correspond to molybdenum and sulfur atoms, respectively. f) The first two charge/discharge curves and g) cycling performance of pure Mg_2_Mo_6_S_8_ cathode at 20 mA g^−1^ and room temperature (RT).

Whereas, cathode premagnesiation additives have relatively high open circuit voltage and chemical stability and can be readily integrated with the existing electrode preparation technologies,^[^ ^14^, ^16^ ^]^ guaranteeing their application in our anode‐free RMBs. Finding an appropriate cathode premagnesiation additive is a prerequisite to construct a high‐energy‐density AFRMBs. Among the commonly used Mg‐containing compounds (**Figure** **2**a), MgS is deemed as one of the most promising premagnesiation additives in consideration of its high theoretical “donor” Mg‐ion capacity of 950 mAh g^−1^, good compatibility with the current battery fabrication processes including slurry fabrication, electrode drying, and battery assembly, and simple and straightforward materials processing with low cost and high safety. More importantly, with the lowest formation energy, MgS can be more easily decomposed to extract Mg with low overpotentials,^[^ ^17^ ^]^ and therefore is applied as the cathode premagnesiation additive in this work. In contrast, MgO additive, despite high theoretical capacity of 1330 mAh g^−1^, is excluded due to the large decomposition hysteresis and voltage mismatch with commonly used cathodes of Mg batteries (<2.5 V).^[^ ^18^ ^]^

Besides MgS cathode premagnesiation additive, magnesiated Chevrel phase Mg_2_Mo_6_S_8_, known as the model cathode of Mg batteries, is adopted as the main cathode material in this work because of its fast magnesiation kinetics and excellent cycling stability.^[^ ^11^, ^19^ ^]^ Mo_6_S_8_ nanosheet is first synthesized using our previously reported method.^[^ ^11h^ ^]^ Its X‐ray diffraction (XRD) patterns well match the standard power diffraction file (PDF) No. 89‐5114 belonging to a rhombohedral structure with the *R3̅* space group (Figure 2b).^[^ ^20^ ^]^ The lateral length of Mo_6_S_8_ nanosheets reaches several micrometers with the thickness of tens of nanometers (Figure 2c and Figure S5, Supporting Information). Mo_6_S_8_ nanosheets are single‐crystalline with a (101) rhombohedral lattice spacing of 6.43 Å, revealed by HRTEM and corresponding FFT (Figure 2d). Spherical aberration‐corrected scanning transmission electron microscopy (STEM) is also employed to obtain a direct vision of the atomic structure, in which six molybdenum atoms and eight sulfur atoms can be clearly observed in the [211] direction of the low‐angle annular dark‐field (LAADF) image (Figure 2e). To obtain Chevrel phase Mg_2_Mo_6_S_8_, Mo_6_S_8_ nanosheet is chemically premagnesiated by immersing it into the 0.5 m di‐*n*‐butyl magnesium/heptane solution for 7 days. The Mg_2_Mo_6_S_8_ cathode material delivers a capacity of 114.6 mAh g^−1^ in the first charge process (Figure 2f), indicating that 1.89 Mg^2+^ is intercalated into Mo_6_S_8_ during chemical premagnesiation process. Except for the first cycle, no obvious capacity decay is observed over 100 cycles, denoting the highly stable cycling performance of Mg_2_Mo_6_S_8_ cathode (Figure 2g).

**Figure 3:**
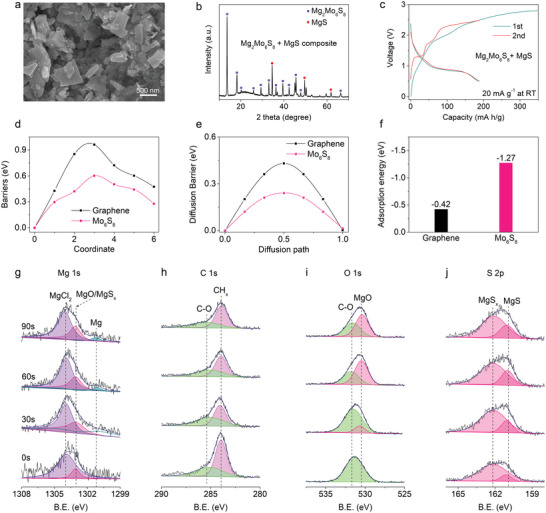
Electrochemical activation of Mo_6_S_8_ on MgS and interface chemistry of Mg metal anode in anode‐free Mg_2_Mo_6_S_8_‐MgS/Cu battery. a) SEM and b) XRD of Mg_2_Mo_6_S_8_‐MgS composite cathode. c) The first two charge/discharge curves of anode‐free Mg_2_Mo_6_S_8_‐MgS/Cu battery at 20 mA g^−1^ within 0.5–2.8 V for the 1st cycle and 0.5–2.5 V for the 2nd cycle. The specific capacities are calculated based on the mass of Mg_2_Mo_6_S_8_ only. d) Energy profiles of the decomposition of MgS cluster on the Mo_6_S_8_ (101) and graphene surface. e) Energy profiles of Mg‐ion diffusion processes in Mo_6_S_8_ and graphene at dilute Mg concentrations. f) Binding energy of MgS_6_ species adsorption on Mo_6_S_8_ and graphene surface. High‐resolution XPS spectra of g) Mg 1s, h) C 1s, i) O 1s, and j) S 2p for Mg metal deposited in anode‐free Mg_2_Mo_6_S_8_‐MgS/Cu batteries in MACC electrolyte after various etching time: 0, 30, 60, and 90 s.

The anode‐free rechargeable Mg battery is constructed using Mg_2_Mo_6_S_8_ and MgS (4:1 by molar ratio) as the composite cathode, a bare Cu current collect on the anode side, and MACC electrolyte. Mg_2_Mo_6_S_8_ and MgS are ball‐milling mixed to obtain the composite cathode, in which Mg_2_Mo_6_S_8_ and MgS are uniformly distributed (**Figure** **3**a). XRD patterns show clear signals of Mg_2_Mo_6_S_8_ and MgS (Figure 3b), indicating that their structures are well maintained after ball‐milling. The electrochemical mechanism of anode‐free Mg_2_Mo_6_S_8_‐MgS/Cu battery is investigated, in which the battery is first charged to 2.8 V to trigger the decomposition of MgS and generate more Mg plated on the Cu current collector of anode side (Figure 3c). During the charge process, MgS might be decomposed to be polysulfides MgS_6_ or elemental sulfur.^[^ ^21^ ^]^ The capacity of 350 mAh g^−1^ is delivered in the first charge process, in which some parasitic reactions might contribute to the charge capacity in consideration of the high voltage cutoff of 2.8 V. 112 mAh g^−1^ below 2.0 V can be attributed to Mg_2_Mo_6_S_8_,^[^ ^11h^ ^]^ while 238 mAh g^−1^ between 2.0 and 2.8 V is mainly ascribed to the decomposition of MgS.^[^ ^17a^ ^]^ In the first discharge process, the battery partially reversibly obtains the capacity of 190 mAh g^−1^. The irreversible capacity of 160 mAh g^−1^ in the first cycle corresponds to the irreversible Mg left on the anode side, which will replenish fresh Mg to compensate for Mg loss during the following cycles. In the second cycle, the capacity of 185 mAh g^−1^ is reversible with CE above 99%. Ruling out the contribution from Mg_2_Mo_6_S_8_, MgS in the Mg_2_Mo_6_S_8_‐MgS composite cathode is calculated to deliver the capacity of 415 mAh g^−1^. In contrast, pure MgS cathode only affords the capacity of 173 mAh g^−1^ when charged to 2.8 V (Figure S9, Supporting Information), much less than that in Mg_2_Mo_6_S_8_‐MgS composite cathode, indicating that Mg_2_Mo_6_S_8_ might promote the decomposition of MgS.

Density functional theory calculations are performed to pin down the interplay between MgS and Mg_2_Mo_6_S_8_ in the composite cathode. The decomposition energy barrier of MgS is calculated to evaluate its demagnesiation reaction kinetics on the surface of Mo_6_S_8_ and graphene. The MgS decomposition process mainly involves one Mg atom dissociating from MgS and diffusing away from S atoms, accompanied by the breaking of Mg—S bonds. The decomposition barrier of MgS on the Mo_6_S_8_ (101) surface (0.60 eV) is much lower than that in the graphene substrate (0.96 eV) (Figure 3d and Figures S10 and S11, Supporting Information), demonstrating the excellent catalytic effect of Mo_6_S_8_ on the decomposition of MgS. Additionally, the MgS conversion chemistry involves Mg^2+^ diffusion in the Mo_6_S_8_ necessitating the calculation of Mg^2+^ diffusion barriers at the Mo_6_S_8_ and graphene surface (Figure 3f and Figure S12, Supporting Information). The much lower barrier of Mg ions in Mo_6_S_8_ (0.24 eV) than that at the graphene surface (0.43 eV) induces a higher diffusion rate, which will promote the decomposition of MgS. Besides the electrocatalysis, the adsorption effect imposed by Mo_6_S_8_ on the polysulfides MgS_6_ that is the intermediate of MgS conversion is uncovered, in which Mo_6_S_8_ (101) exhibits much higher adsorption energy (−1.27 eV) (Figure 3e and Figure S13, Supporting Information) than graphene (−0.42 eV). The strong adsorption by Mo_6_S_8_ will restrain the dissolution of MgS_6_ in favor of the stable cycle of Mg_2_Mo_6_S_8_‐MgS composite cathode.

In addition to the interplay between Mg_2_Mo_6_S_8_ and MgS in the cathode, the interface chemistry of Mg metal anode also dictates the electrochemical performance of anode‐free Mg batteries. X‐ray photoelectron spectroscopy (XPS) spectra of Mg metal deposited in anode‐free Mg_2_Mo_6_S_8_‐MgS/Cu batteries are collected to elaborate on the interface chemistry (Figure 3g–j and Figures S14–S16, Supporting Information). The Mg 1s spectra are deconvoluted into three peaks that can be assigned to the MgCl_2_ (1304 eV),^[^ ^22^ ^]^ MgO/MgS*_x_* (1303 eV),^[^ ^23^ ^]^ and Mg^0^ (1301.2 eV)^[^ ^17^, ^23^ ^]^ (Figure 3g). The strong MgCl_2_ signals in Mg 1s and Cl 2p (198.8 eV)^[^ ^24^ ^]^ throughout the etching time indicate that MgCl_2_ is not just absorbed on the surface but also exists as a component of SEI film (Figure 3g and Figures S14 and S15, Supporting Information). Mg 1s peaks at 1303 eV are crosschecked with O 1s peaks at 530.5 eV^[^ ^25^ ^]^ (Figure 3i) to show the presence of MgO that comes from the reduction of electrolyte. Inorganic MgO signals are enhanced with etching time accompanied with the receding organic C—O signals (O 1s at 531.7 eV and C 1s at 285.4 eV) (Figure 3h and Figure S16, Supporting Information), indicating that organic compounds reside on top of the inorganic species. The MgS*_x_* (162.2 eV)^[^ ^21b^ ^]^ and MgS (160.9 eV)^[^ ^23b^ ^]^ peaks in S 2p spectra evidence the reduction of dissolved polysulfides on Mg anode (Figure 3j). The intensity of MgS signals enhances with etching time, probably due to the deep reduction of sulfur species near Mg metal. During cycling, magnesium polysulfides generated from Mg_2_Mo_6_S_8_‐MgS cathode will dissolve into the MACC electrolyte. When the polysulfide‐containing electrolyte is brought to the vicinity of Mg anode, it will be reduced to form SEI until the kinetic stability is reached. According to the XPS results, the SEI film of Mg anode mainly consists of three layers: an inorganic layer containing multiple Mg compounds (MgCl_2_, MgO, MgS, etc.), a middle layer containing organic components (C—O etc.), and an adsorption layer (CH*_x_*, MgCl_2_, etc.). The inorganic layer of SEI film is not compact and formed by complex compositions. Mg^2+^ can migrate through the interspace and grain boundary to maintain the high reversibility of Mg metal anode. The Mg^2+^‐conductive and electron‐insulative SEI will provide protection to Mg metal from the constant corrosion by electrolyte and thus promote the cycling stability of anode‐free Mg_2_Mo_6_S_8_‐MgS/Cu batteries.

**Figure 4:**
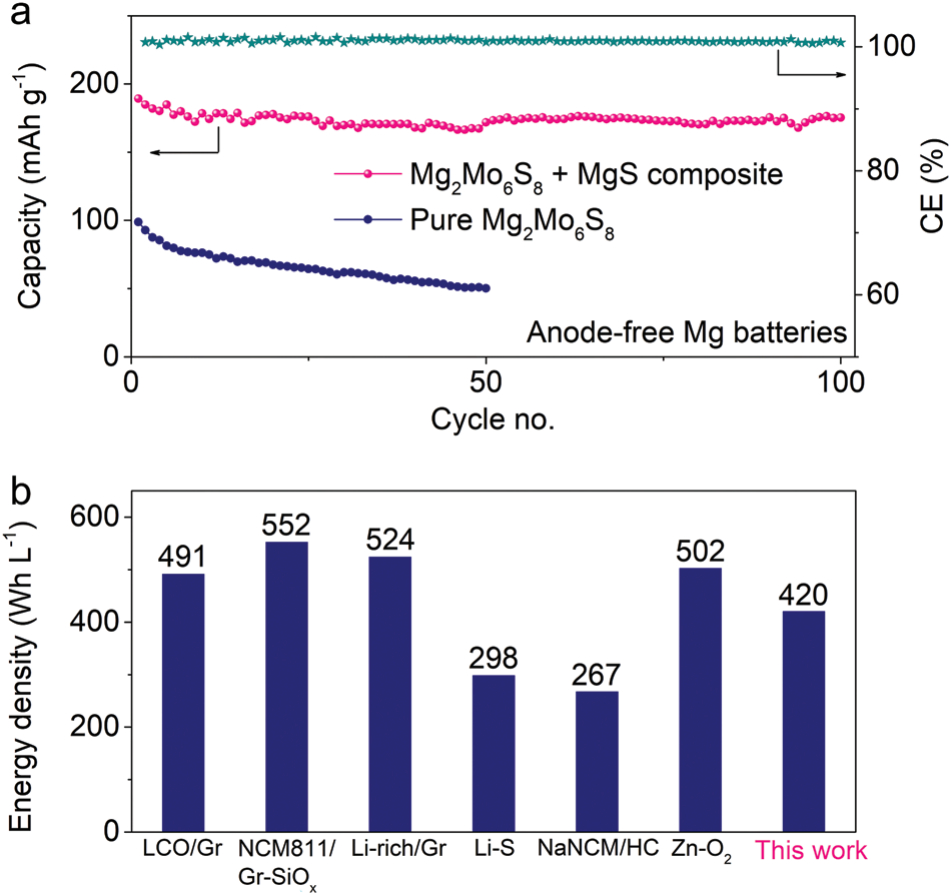
Electrochemical performance of anode‐free Mg_2_Mo_6_S_8_‐MgS/Cu batteries. a) Long‐term cycling stability for the first 100 cycles in MACC electrolyte at 20 mA g^−1^. b) Comparison on energy densities of anode‐free Mg_2_Mo_6_S_8_‐MgS/Cu battery with other battery systems calculated applying industrial parameters. The specific capacities are calculated based on the mass of Mg_2_Mo_6_S_8_ only.

The long‐term cycling stability of anode‐free Mg_2_Mo_6_S_8_‐MgS/Cu batteries is evaluated in MACC electrolyte at 20 mA g^−1^, in which the capacity of 175 mAh g^−1^ remains after 100 cycles corresponding to the 92% capacity retention (**Figure** **4**a). In contrast, anode‐free batteries with other molar ratios (1:0 and 2:1) deliver lower capacities with lower capacity retention (Figure 4a and Figure S17, Supporting Information). The much higher capacity delivery and retention highlight the advantage of our Mg_2_Mo_6_S_8_‐MgS composite cathode. On one hand, MgS as the premagnesiation additive constantly decomposes during cycling to replenish fresh Mg for Mg loss caused by the electrolyte corrosion. On the other hand, both Mg_2_Mo_6_S_8_ and MgS act as the active material to reversibly provide high capacities. In view of zero‐excess Mg metal in the anode‐free Mg_2_Mo_6_S_8_‐MgS/Cu batteries, the volume of battery is minimized with the energy density maximized. Adopting the industrial parameters of multilayer pouch cells (Table S1, Supporting Information), the cell‐level energy density of our anode‐free Mg batteries is calculated to be 420 Wh L^−1^, which is highly competitive among the most pervasive battery systems (Figure 4b).

## 2 Conclusion

In this work, a proof‐of‐concept of anode‐free Mg batteries is rationally designed using Mg_2_Mo_6_S_8_‐MgS composite cathode, in which MgS as the premagnesiation additive constantly decomposes over cycling to replenish fresh Mg for Mg loss caused by electrolyte corrosion. Both Mg_2_Mo_6_S_8_ and MgS act as the active material to reversibly provide high capacities. In the composite cathode, Mg_2_Mo_6_S_8_ catalyzes the decomposition of MgS to release more Mg inventory and lower the conversion hysteresis. Also, Mg_2_Mo_6_S_8_ provides the strong adsorption to the polysulfides to restrain their dissolution contributing to the stable cycle. Besides, the Mg^2+^‐conductive and electron‐insulative SEI formed in anode‐free Mg_2_Mo_6_S_8_‐MgS/Cu batteries protects Mg metal from the constant corrosion by electrolyte and thus promotes the cycling performance. Accordingly, the anode‐free Mg_2_Mo_6_S_8_‐MgS/Cu batteries deliver a reversible capacity of 190 mAh g^−1^ with the capacity retention of 92% after 100 cycles. The energy density of our anode‐free Mg battery is calculated to be highly competitive 420 Wh L^−1^. With more discovery of high‐performance cathodes and electrolytes, the volumetric energy density will be further strengthened to promise the practicable application of Mg batteries. This work will act as a primer to ignite the enormous prospective researches on the anode‐free Mg batteries.

## 3 Experimental Section

Material synthesis, material characterizations, electrochemical measurements, and calculation details are provided in the Supporting Information.

## Conflict of Interest

The authors declare no conflict of interest.

## Supporting information

Supporting Information

## Data Availability

The data that support the findings of this study are available from the corresponding author upon reasonable request.
