# Supplementary material for: The Proof‐of‐Concept of Anode‐Free Rechargeable Mg Batteries
Source: Adv Sci (Weinh). 2023 Mar 20;10(14):2207563. doi: 10.1002/advs.202207563 (PMC10190628; doi:10.1002/advs.202207563)
Supplement: Supplementary file 1 — Supporting Information [file ADVS-10-2207563-s001.pdf]

The proof-of-concept of anode-free rechargeable Mg batteries

Minglei Mao,*[1] Xueru Fan,[1] Wei Xie,[1] Haoxiang Wang,[1] Liumin Suo,*[3] and Chengliang Wang*[1,2]

[1]School of Integrated Circuits, School of Optical and Electronic Information, Wuhan National Laboratory for Optoelectronics (WNLO), Huazhong University of Science and Technology, Wuhan 430074, China

[2]Wenzhou Advanced Manufacturing Technology Research Institute, Huazhong University of Science and Technology, Wenzhou 325035, China

[3]Beijing Advanced Innovation Center for Materials Genome Engineering, Institute of Physics, Chinese Academy of Sciences/Beijing National Laboratory for Condensed Matter Physics, Beijing 100190, China

*Email: mlmao@hust.edu.cn, suoliumin@iphy.ac.cn, clwang@hust.edu.cn

Experimental Section

Material synthesis

Synthesis of Chevrel phase Mo_6S_8. Chevrel phase Mo_6S_8 is synthesized according to our previously reported method.[1] Specifically, MoS_2, Cu, and Mo powders were ball-mixing mixed and pressed into pellets with some iodine. After sealed in a swagelok stainless steel vessel, the mixture was gradually heated to 800 ºC and kept for 24 h under Ar to get $Cu_2Mo_6S_8$. Mo_6S_8 powder was obtain by leaching out Cu from the as-prepared $Cu_2Mo_6S_8$ precursor in a 6 M HCl solution with oxygen bubbling.

$Mg_2Mo_6S_8$ was obtained by immersing Mo_6S_8 into the 0.5 M di-n-butylmagnesium in heptane solution for 7 days and rinsing for 3 times using heptane, which is learned from the reported chemical lithiation process using n-butyllithium in heptane.[2-4] The duration of 7 days is applied to guarantee the complete chemical pre-magnesiation of Mo_6S_8 nanosheets in consideration of the slow magnesiation kinetics. $Mg_2Mo_6S_8$ and MgS were mixed at a weight ratio of 4:1 by ball-milling at 300 rpm for 2 h under Ar to form $Mg_2Mo_6S_8$-MgS composite.

Electrochemical measurements

Cell assembly was carried out in an Ar-filled glovebox with O_2 and H_2O levels below 0.1 ppm. The $Mg_2Mo_6S_8$-MgS composite cathode was prepared by pressing the as-prepared composites, ketjen black, and PTFE at a weight ratio of 8:1:1 onto a stainless-steel mesh. Anode-free Mg batteries were assembled using pouch cells with MACC as the electrolyte, Cu foil as the current collector of anode, and Whatman glass fibers as separators. The electrochemical test was conducted on a LAND-CT2001A battery test station (LAND Electronic Co.) with a voltage cutoff of 0.5−2.5 V at room temperature (RT). The specific capacities are calculated based on the mass of $Mg_2Mo_6S_8$ only unless otherwise stated. Nyquist plots were recorded using Autolab PGSTAT302N (Metrohm, Switzerland) at a frequency range of 0.01−100 kHz. The capacity throughout the paper is calculated based on the mass of $Mg_2Mo_6S_8$-MgS samples.

Material characterizations

The XRD patterns were measured using Cu Kα radiation on an X'Pert Pro MPD X-ray diffractometer from 10° to 80° (2θ). The morphologies of samples were investigated by scanning electron microscopy (Hitachi S-4800), transmission electron microscopy (JEM 2100Plus, JEOL Limited Corporation, Japan), and advanced spherical aberration-corrected scanning transmission electron microscopy combined with low-angle annular dark field as well as annular bright field (JEM-ARM200CF, JEOL, Tokyo, Japan). The X-ray photoelectron spectroscopy (XPS) spectra were recorded with a spectrometer having Mg/Al Kα radiation (ESCALAB 250 Xi, Thermo Fisher). All binding energies were corrected using the signal of carbon at 284.8 eV as an internal standard. For ex situ XPS measurements, pouch cells were disassembled in an argon-filled glovebox and the electrodes were washed in tetrahydrofuran (anhydrous, Alfa Aesar, 99.9%) for three times to remove the electrolyte, and then the dry samples were obtained and transferred to the machine with an argon-filled sealing tube as a transfer box. In this process, all samples were exposed to air within 3−4 s.

Theoretical calculations

The Vienna Ab Initio Package (VASP) was employed to perform all the density

functional theory (DFT) calculations within the generalized gradient approximation (GGA) using the Perdew, Burke, and Enzerhof (PBE) formulation.[5-7] The projected augmented wave (PAW) potentials were applied to describe the ionic cores and take valence electrons into account using a plane wave basis set with a kinetic energy cutoff of 450 eV.[8, 9] Partial occupancies of the Kohn–Sham orbitals were allowed using the Gaussian smearing method and a width of 0.05 eV. The electronic energy was considered self-consistent when the energy change was smaller than 10^{-4} eV. A geometry optimization was considered convergent when the force change was smaller than 0.04 eV/Å. Grimme's DFT-D3 methodology was used to describe the dispersion interactions.[10] The equilibrium lattice constants of unit cell were optimized when using a 2×2×1 Monkhorst-Pack k-point grid for Brillouin zone sampling. The Climbing Image-Nudged Elastic Band methods were employed to calculate the Mg ions migration barriers in the structures. Finally, the adsorption energies (E_{ads}) were calculated as $E_{ads} = E_{ad/sub} - E_{ad} - E_{sub}$, where $E_{ad/sub}$, E_{ad}, and E_{sub} are the total energies of the optimized adsorbate/substrate system, the adsorbate in the structure, and the clean substrate, respectively.

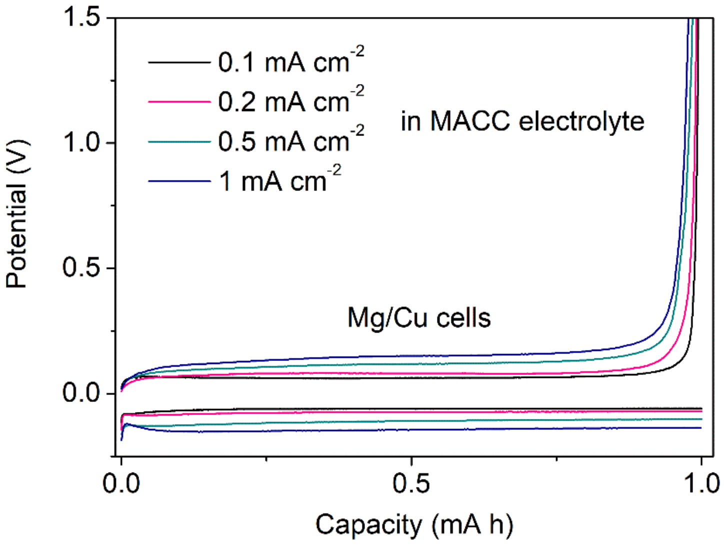

Figure S1. The typical discharge/charge curves for Mg stripping and deposition process in Mg/Cu half cells for 1 mAh cm^{-2} at various current densities.

The typical discharge/charge curves show that Mg plating and stripping process has a high CE of 99.4% at 0.1 mA cm^{-2} with a small hysteresis of 0.05 V in Mg/Cu half cells. With the increase of current densities, the CE has a margin decrease to 99% with

a slightly higher hysteresis of 0.25 V at 1 mA cm^{-2}.

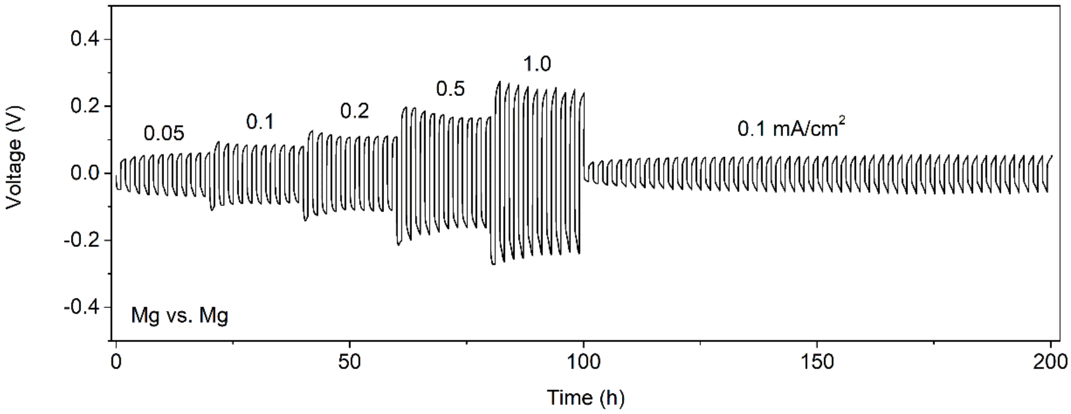

Figure S2. Voltage profiles of galvanostatic Mg plating/stripping in the Mg/Mg symmetric cells at various current densities for 2h at each step.

During long-term cycling process in Mg/Mg symmetric cells, Mg metal can support a 200h cycle with 1h plating and stripping process, indicating that no dendrite is formed to cause the short-circuit of cells. The hysteresis climbs with the increase of current densities. After 100h-activation process, the hysteresis is lessened to less than 100 mV when the current density returns to 0.1 mA cm^{-2}.

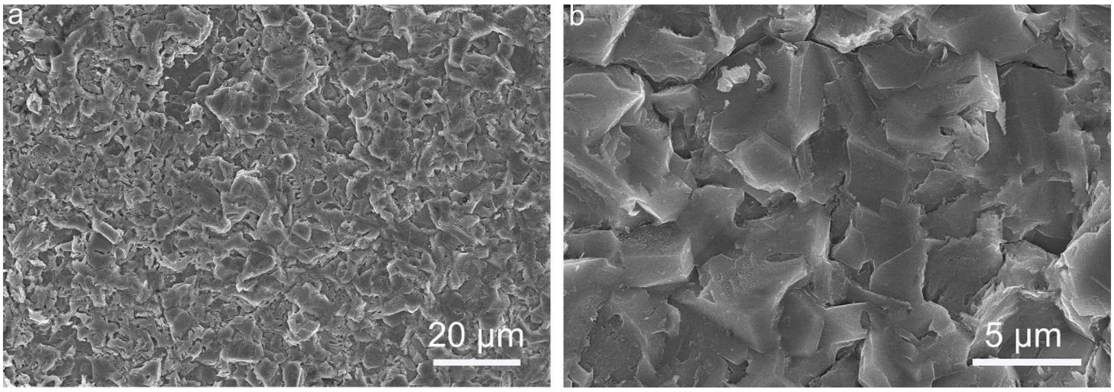

Figure S3. (a) and (b) SEM images of Mg deposits in MACC electrolyte for 1 mAh cm^{-2} at 0.5 mA cm^{-2} in Mg/Cu cells.

SEM images show that Mg deposits exhibit hexagon morphology without any dendrite observed.

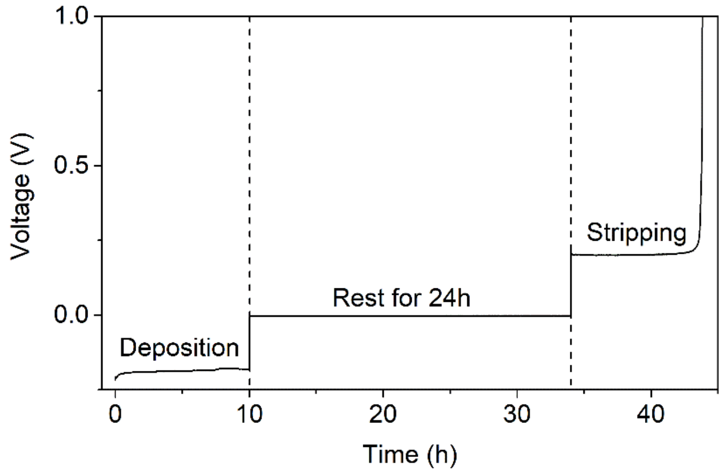

Figure S4. The typical curves for Mg deposition and stripping process in Mg/Cu half cells for 1 mAh cm^{-2} at 0.5 mA cm^{-2} with 24 h interval.

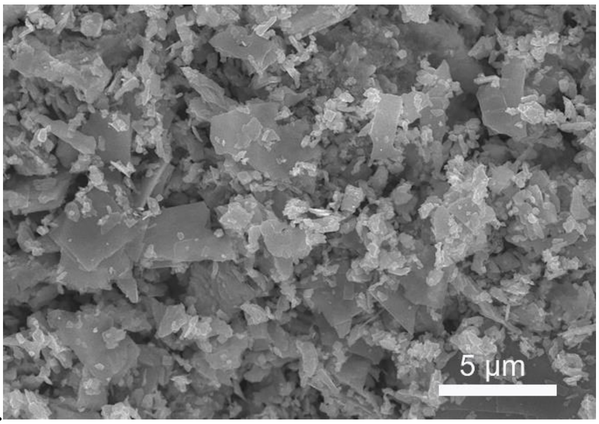

Figure S5. SEM image of Chevrel phase Mo_6S_8 nanosheets.

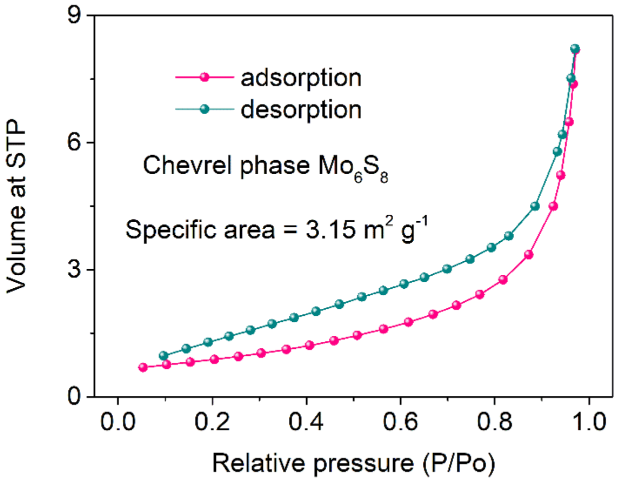

Figure S6. BET nitrogen adsorption and desorption isotherms of Chevrel phase Mo_6S_8. The specific

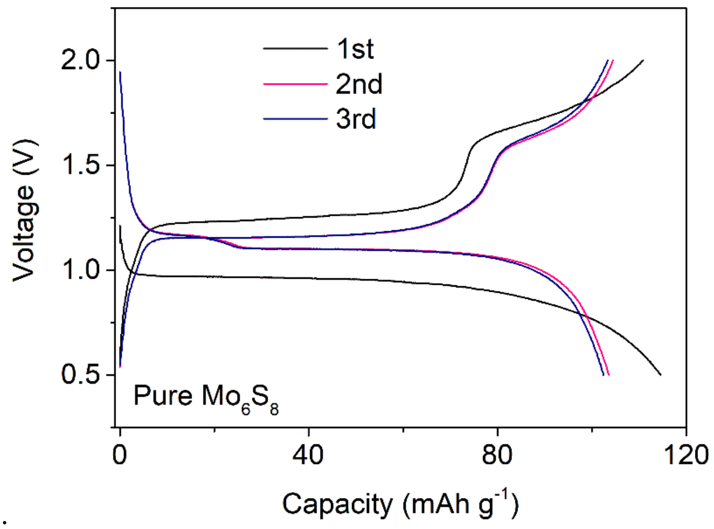

area of Mo_6S_8 is 3.15 m^2 g^{-1}.

Figure S7. The first three voltage curves of pure Mo_6S_8 cathode at 20 mA g^{-1}

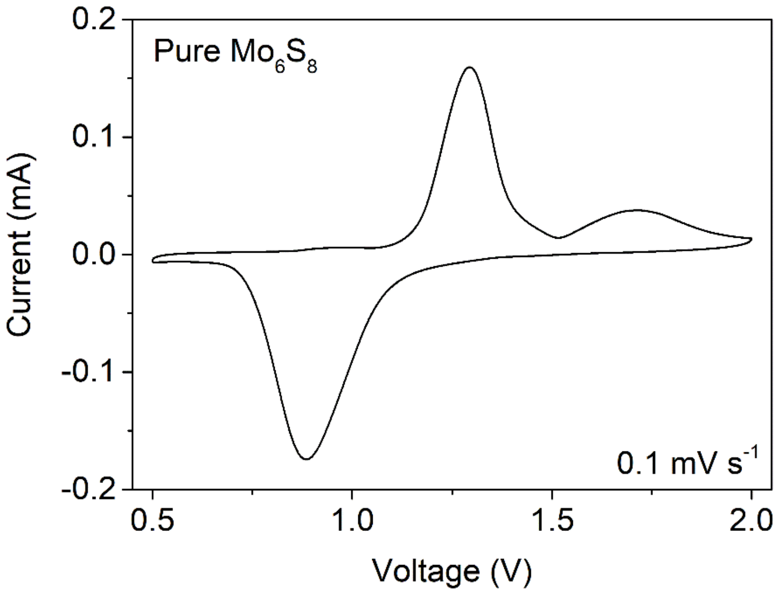

Figure S8. Cyclic voltammetry curves of pure Mo_6S_8 cathode at a scan rate of 0.1 mV s^{-1}.

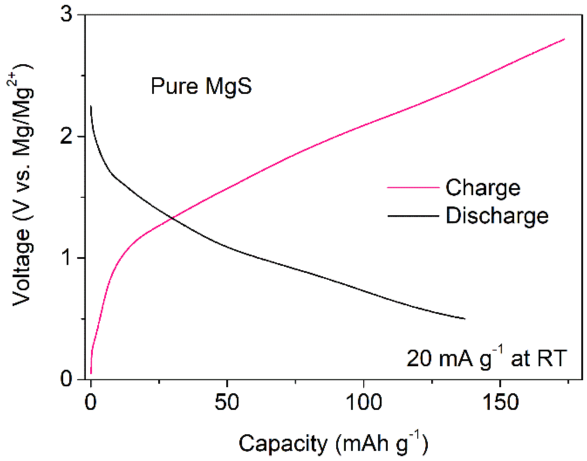

Figure S9. The first charge/discharge curves of pure MgS between 0.5 and 2.8 V at 20 mA g^{-1} and

RT. The specific capacities are calculated based on the mass of MgS.

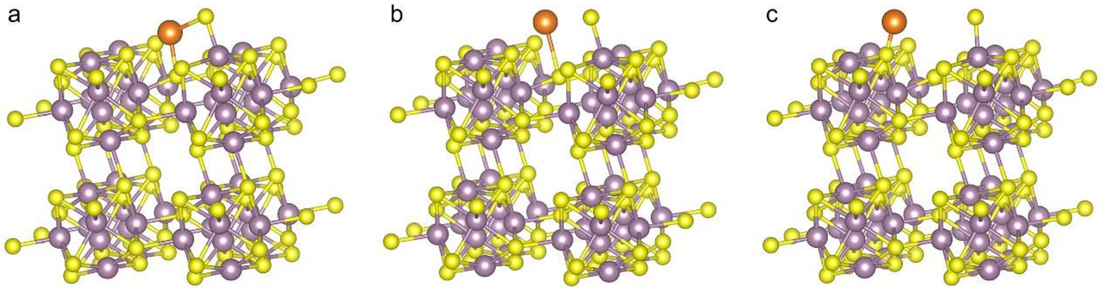

Figure S10. The optimized schematic representations of decomposition pathways of MgS on the Mo_6S_8 (101) surface. Beige, yellow, and wine balls symbolize molybdenum, sulfur, and magnesium atoms, respectively.

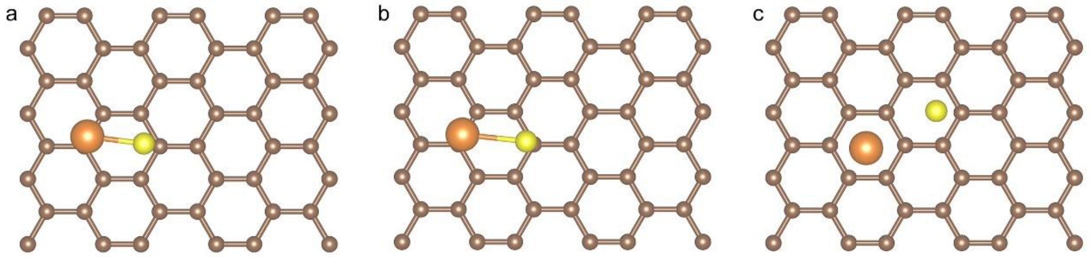

Figure S11. The optimized schematic representations of decomposition pathways of MgS on the graphene surface. Brown, yellow, and wine balls symbolize carbon, sulfur, and magnesium atoms, respectively.

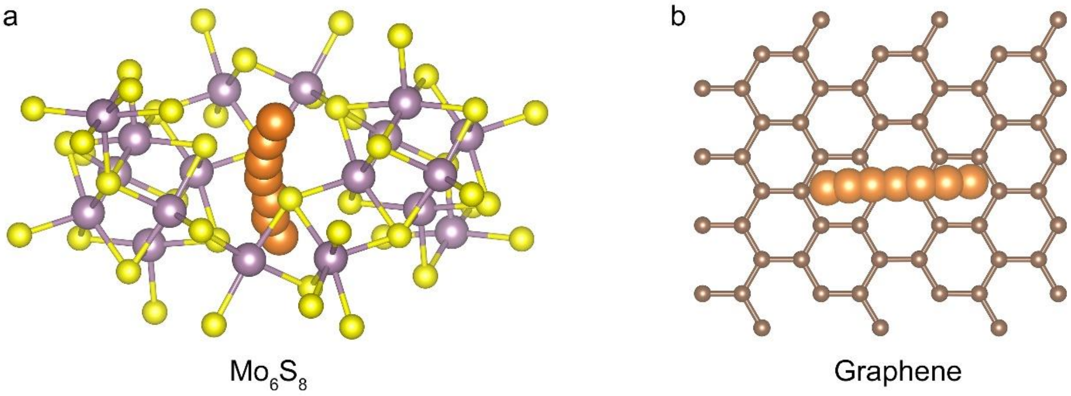

Figure S12. The relaxed trajectory for Mg^{2+} hopping in (a) Chevrel phase Mo_6S_8 and (b) graphene at dilute Mg concentrations. Beige, yellow, brown, and wine balls symbolize molybdenum, sulfur, carbon, and magnesium atoms, respectively.

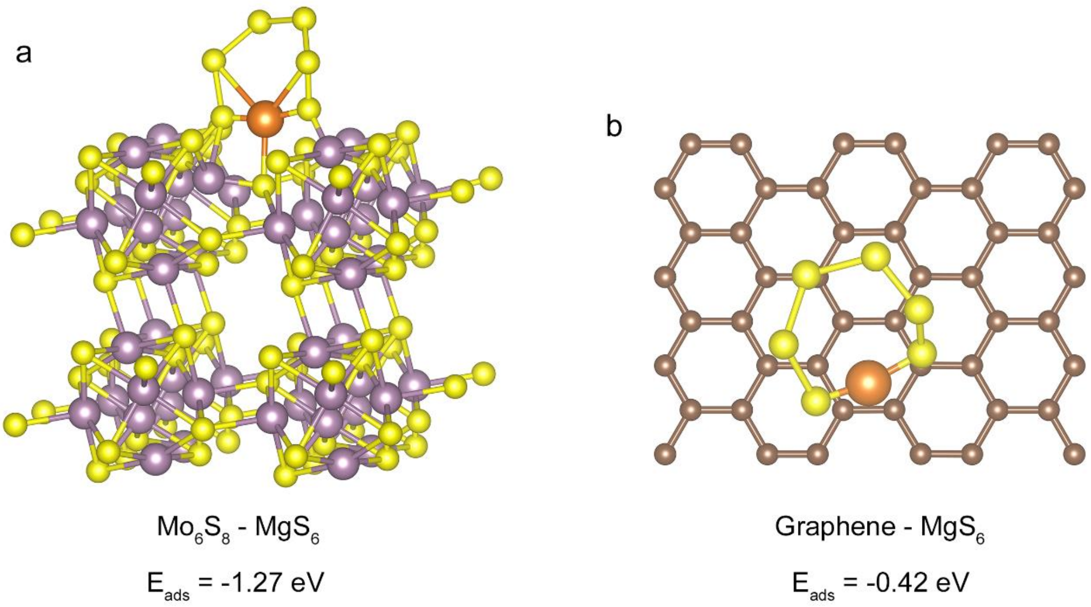

Figure S13. Atomic conformation and binding energy for MgS_6 species adsorption on (a) Chevrel phase Mo_6S_8 and (b) graphene surface, respectively. Beige, yellow, brown, and wine balls symbolize molybdenum, sulfur, carbon, and magnesium atoms, respectively.

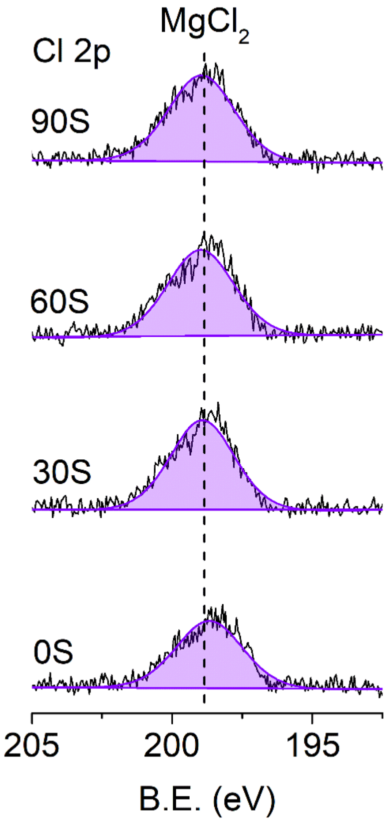

Figure S14. High-resolution XPS Cl 2p spectra of Mg anode after various etching time

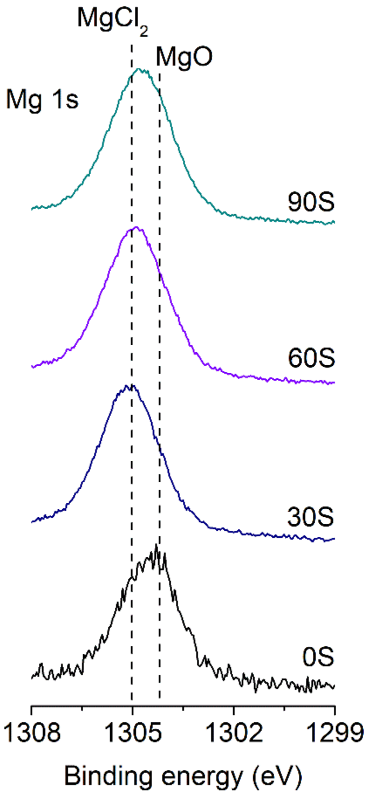

Figure S15. High-resolution XPS Mg 1s spectra of Mg metal deposited in MACC after various etching time.

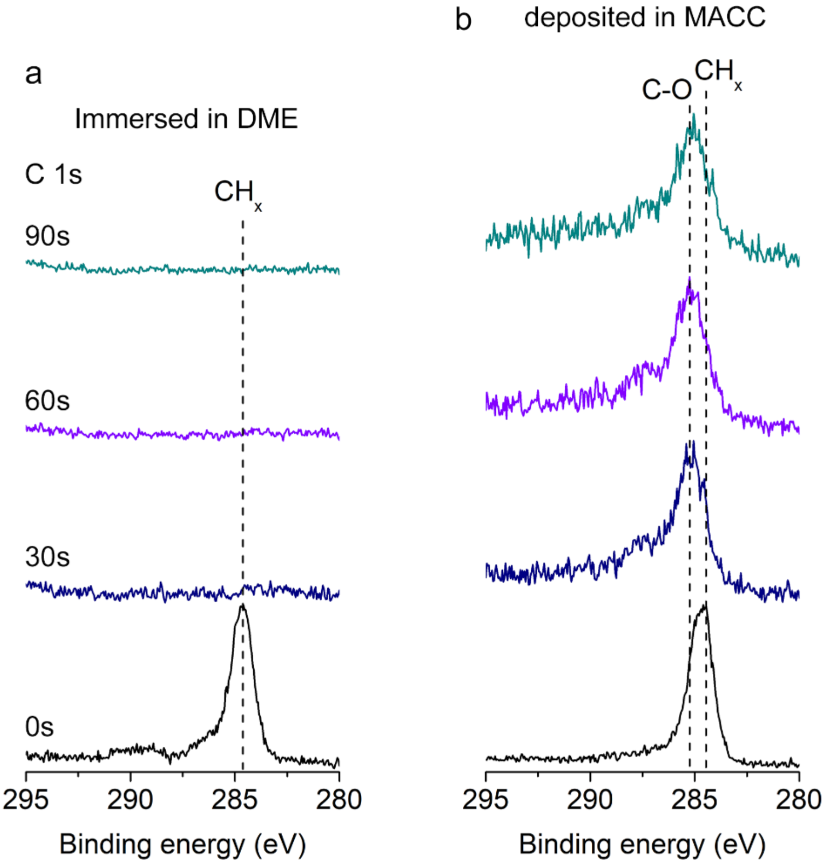

Figure S16. High-resolution XPS C1s spectra of Mg metal: (a) immersed in DME and (b) deposited

in MACC electrolyte after various etching time.

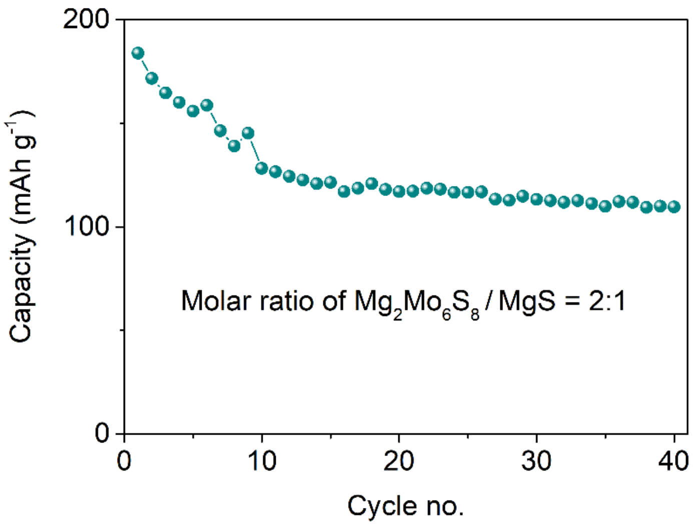

Figure S17. Long-term cycling stability of anode-free $Mg_2Mo_6S_8$-MgS/Cu batteries with the molar ratio of 2:1 for the first 40 cycles in MACC electrolyte at 20 mA g^{-1}.

Table S1. The specifications of the anode-free $Mg_2Mo_6S_8$-MgS/Cu pouch cells using industrial parameters to calculate their energy density.

	Specification	Value
Cathode ($Mg_2Mo_6S_8$ + MgS)	Capacity	190.3 mAh g^{-1}
	Voltage	1 V
	Ratio of active materials	94 wt%
	Density (active materials + binder + carbon)	2.93 g cm^{-3}
	Loading	18 mg cm^{-2}
	Thickness (both side coating)	130.7 μm
	Number of stacks	49
Separator	Thickness	16 μm
	Number of stacks	49
Anode	Thickness (Cu foils)	6 μm
	Number of stacks	50
Full cells	Dimension	300 * 100 * 7.489 mm^3
	n/p ratio	1
	Total capacity	94.5 Ah
	Voltage	1 V
	Energy density	420 Wh L^{-1}

The thickness of pouch cells includes cathode, anode, current collector, and separators without taking the electrolyte volume into account.

References

1. Mao, M.; Lin, Z.; Tong, Y.; Yue, J.; Zhao, C.; Lu, J.; Zhang, Q.; Gu, L.; Suo, L.; Hu, Y.-S.; Li, H.; Huang, X.; Chen, L., Iodine Vapor Transport-Triggered Preferential Growth of Chevrel Mo6S8 Nanosheets for Advanced Multivalent Batteries. *ACS Nano* **2020,** *14* (1), 1102-1110.
2. Dines, M. B., Lithium intercalation via n-Butyllithium of the layered transition metal dichalcogenides. *Materials Research Bulletin* **1975,** *10* (4), 287-291.
3. Peramunage, D.; Abraham, K. M., Preparation and Electrochemical Characterization of Overlithiated Spinel LiMn2 O 4. *Journal of The Electrochemical Society* **1998,** *145* (4), 1131.
4. Aravindan, V.; Lee, Y.-S.; Madhavi, S., Best Practices for Mitigating Irreversible Capacity Loss of Negative Electrodes in Li-Ion Batteries. *Advanced Energy Materials* **2017,** *7* (17), 1602607.
5. Kresse, G.; Furthmüller, J., Efficient iterative schemes for ab initio total-energy calculations using a plane-wave basis set. *Physical Review B* **1996,** *54* (16), 11169-11186.
6. Perdew, J. P.; Burke, K.; Ernzerhof, M., Generalized Gradient Approximation Made Simple. *Physical Review Letters* **1996,** *77* (18), 3865-3868.
7. Kresse, G.; Joubert, D., From ultrasoft pseudopotentials to the projector augmented-wave method. *Physical Review B* **1999,** *59* (3), 1758-1775.
8. Blöchl, P. E., Projector augmented-wave method. *Physical Review B* **1994,** *50* (24), 17953-17979.
9. Grimme, S.; Antony, J.; Ehrlich, S.; Krieg, H., A consistent and accurate ab initio parametrization of density functional dispersion correction (DFT-D) for the 94 elements H-Pu. *The Journal of Chemical Physics* **2010,** *132* (15), 154104.
10. Henkelman, G.; Uberuaga, B. P.; Jónsson, H., A climbing image nudged elastic band method for finding saddle points and minimum energy paths. *The Journal of Chemical Physics* **2000,** *113* (22), 9901-9904.
